# Adding Pluggable and Personalized Natural Control Capabilities to Existing Applications

**DOI:** 10.3390/s150202832

**Published:** 2015-01-28

**Authors:** Fabrizio Lamberti, Andrea Sanna, Gilles Carlevaris, Claudio Demartini

**Affiliations:** Dipartimento di Automatica e Informatica, Politecnico di Torino, Corso Duca degli Abruzzi 24, 10129 Torino, Italy; E-Mails: andrea.sanna@polito.it (A.S.); gilles.carlevaris@polito.it (G.C.); claudio.demartini@polito.it (C.D.)

**Keywords:** natural user interfaces, poses, gestures and voice, human-computer interaction

## Abstract

Advancements in input device and sensor technologies led to the evolution of the traditional human-machine interaction paradigm based on the mouse and keyboard. Touch-, gesture- and voice-based interfaces are integrated today in a variety of applications running on consumer devices (e.g., gaming consoles and smartphones). However, to allow existing applications running on desktop computers to utilize natural interaction, significant re-design and re-coding efforts may be required. In this paper, a framework designed to transparently add multi-modal interaction capabilities to applications to which users are accustomed is presented. Experimental observations confirmed the effectiveness of the proposed framework and led to a classification of those applications that could benefit more from the availability of natural interaction modalities.

## 1. Introduction

Gestures and sound play a key role in human-to-human communications, because they enable a direct expression of mental concepts [1,2]. A large number of human-computer interaction (HCI) paradigms based on such interaction means have been developed since the 1980s [3,4]. Interfaces based on these paradigms are generally referred to as natural user interfaces (NUIs). There is evidence to support the use of hand and body gestures over traditional methods in many applications, including the navigation of 3D scenes, the browsing of multimedia data, the control of robotic systems and home appliances, *etc.* [5–10].

Indeed, the design of ever more pervasive and natural interaction mechanisms will play an important role in the future of HCI. This trend is today witnessed by the mass-market diffusion of a number of consumer products integrating components generally used to build NUIs, such as touch sensors and depth cameras.

However, although touch/multi-touch interaction is exploited today in a variety of software products and devices, the development of general purpose applications endowed with gestures and voice control capabilities is still in an embryonic phase or has been only partially accepted by end-users. On the one hand, manufacturers and vendors are developing their NUI-based hardware and software, e.g., for controlling television sets, playing video games, interacting with in-car infotainment systems, *etc.*, and, on the other hand, HCI researchers are trying to find new strategies for bringing out the best of such solutions [11–13].

It is reasonable to expect that a further diffusion of such technologies in everyday generic applications will be favored by the growing availability of ever more affordable and powerful sensors, by improvements in gesture and speech recognition technologies, *etc.* Moreover, the release of ever more comprehensive software development frameworks and the evolution of open standards in the field of NUIs will probably contribute to easing the necessary implementation steps and fostering a broader adoption.

Nonetheless, at the present time, a seamless integration of NUI-based interaction in common applications or in the application development workflow is often difficult to observe or achieve. What is missing is a type of flexible software layer able to “augment” the interaction possibilities for (possibly existing) applications, while at the same time limiting (or even avoiding) significant re-design and re-coding efforts, as well as enabling users to re-define their own interaction schemes. This type of flexibility, which should be expected from next generation NUI-based interaction solutions, has been tested in several research prototypes, aimed at showing how to transform the graphical interface of desktop applications into gesture- and/or voice-enabled user interfaces [14–17].

In some cases, the above prototypes can actually work as “pluggable” frameworks, thus easing the integration steps. They generally gather user's interactions via consumer (often gaming) sensors and devices and transform them into suitable (in some cases, also configurable) commands that can be understood by a given application, thus allowing users to control devices in new ways that had not been initially foreseen.

Unfortunately, these solutions are still characterized by a number of constraints. For instance, some solutions set out specific requirements on the characteristics of the application's original graphical user interface (GUI), thus reducing the range of applicability of the underlying approach. Other solutions focus only on a specific type of interaction, e.g., hand gesture-based, body gesture-based, *etc.*, or, for a given interaction, only manage limited and pre-defined sets of poses, gestures or voice inputs. In some approaches, a particular type of sensor is needed, and there are situations where the proposed technique requires more than one sensor. Finally, in some cases, flexibility is achieved by moving the complexity into the software layer that the developer is supposed to use to write his/her own NUI-based application.

Although, in principle, it could be possible to design a natural interface for any type of application, an important coding step might be required.

In this work, a framework that allows existing applications to be enriched with NUI-based control possibilities without any code re-writing is presented. To reach this goal, the devised framework relies on a description of the selected application's GUI, containing a structured overview of its constituting graphical elements. Such a description can either be made available in the application development phase, obtained by *ad hoc* image processing-based techniques or even generated manually. Customizable mapping rules can then be defined to link a given user's pose, gesture or voice command (or a sequence of them) to one or more specific operating system event, which will then be applied to a particular graphical component of the considered interface to activate it.

The effectiveness of the devised approach has been evaluated by working with a set of poses, gestures and voice inputs captured by the sensors provided by the Microsoft Kinect [18], but the developed framework has been designed to be extended to cover other configurations (for instance, works are currently in progress to integrate the Leap Motion 3D Controller [19]). In this work, experimental tests have been carried out to measure the performances of the recognition modules integrated in the framework, as well as to evaluate the benefits and drawbacks associated with adding new interaction possibilities to existing interfaces. In this way, the classes of applications that could benefit more from the integration of NUI-based interaction capabilities are also identified, and some guidelines for NUI integration are defined.

The remainder of the paper is organized as follows. In Section 2, a brief summary of related works is provided, and key aspects of the technologies exploited in implementing the proposed framework are outlined. In Section 3, the basic idea is presented, and the various elements contributing to the overall architecture are discussed in detail. In Sections 4 and 5, the outcomes of experimental tests that have been carried out to assess system functionality and measure its performances are discussed. Finally, in Section 6, conclusions are drawn, and possible directions for future research in the field are examined.

## 2. Background and Related Technologies

To replace the traditional mouse-keyboard HCI paradigm or to improve its effectiveness, alternative or complementary interaction means able to support the development of possibly more natural human-computer communication protocols may have to be identified. In recent years, many sensing technologies and approaches have been experimented with, including face, eye and gaze recognition and tracking [20,21], marker-less/marker-based tracking [22], voice and touch commands [23], mental stimuli [24], *etc.*

In some cases, the above techniques are exploited for controlling particular applications or specific platforms. This is the case, for instance, of handheld or head-mounted devices used in immersive virtual reality environments [25,26]. In other cases, they are embedded into the particular device (as happens with touch/multi-touch input on smartphones, tablets, *etc.*), and specific support is natively provided by the platform development kit. Lastly, there are cases where a new technology is exploited for controlling various types of consumer products [27].

However, further complexity is introduced when the goal is to extend the reach of such advanced interaction techniques to include common applications running on desktop computers. In this case, when the application has been created by resorting to multi-modal-aware development kits, users can take control of its interface elements, e.g., via embedded accessibility and/or automation callbacks. Unfortunately, when this type of support has not been originally foreseen for the application, integrating natural interaction means could be a task that is hard to accomplish. Important advancements in such directions have been made with the introduction of *ad hoc* development toolkits and the release of standardized interfaces to available sensors, which allow researchers to focus on the more relevant integration and interaction goals.

Thus, a solution allowing users to control existing desktop applications using multi-touch devices was introduced by Paravati *et al.* [14]. A comparable solution, called the Flexible Action and Articulated Skeleton Toolkit (FAAST), tailored to body gestures was proposed by Suma *et al.* [15]. FAAST has been designed to add gesture-based control possibilities to 3D graphics applications and video games running on personal computers. FAAST can interface with RGB-D cameras and extract users' skeleton joint locations and orientations. Pose information can then be used to control virtual avatars in specific 3D environments, play first-person games, *etc.* Moreover, an integrated input emulator is able to translate recognized gestures into mouse and keyboard commands, which can then be exploited to control existing desktop applications (usually other types of 3D graphics software programs, such as virtual reality-based tools). Despite its incredible flexibility, a limitation of such a framework is that applications that the user may want to control need to be designed to support this type of input. Unfortunately, although mouse-based interaction is commonly available in most desktop applications, keyboard shortcuts are not always implemented. In these cases, the FAAST toolkit alone would be of little or no help. Moreover, though gesture translations to operating system events can be customized, with FAAST, the user continues to access applications with the previous paradigm: the presence of a mouse and a keyboard is simply hidden. However, more sophisticated (and possibly more natural) interaction approaches could be originated by totally re-thinking the interaction paradigm and eventually getting rid of such traditional input devices.

A different solution relying on hand gestures was proposed by Wang *et al.* [16]. A technique to perform hand tracking on color images captured by a pair of webcams is used. The approach is designed to track the six degrees of freedom (DOFs) of two hands moving simultaneously in the cameras' field of view. A basic pinch pose is recognized and used as a proxy of various types of user interactions (pointing, selection, *etc.*). The solution proposed is integrated with a dedicated tool for computer-aided design (CAD). The authors continued to work on the original idea by adding other types of sensors (e.g., depth-based) and releasing a software library to be used to endow other applications with pinch-based interaction capabilities. Such a solution is meant to build another layer over vendor-and manufacturer-specific software development kits, with the aim of further easing the deployment of NUI-based applications. Nonetheless, it still requires the developer to make changes to the code of the application that the user may want to control with gestures. Moreover, the possibility to add other poses and gestures and allocate them to specific interaction tasks is not considered.

A preliminary solution designed to partially cope with the above limitations was presented by Lamberti *et al.* [17]. Such a solution starts by considering that, to reach the needed degree of integration and flexibility, a coding-free way for linking elements constituting desktop interfaces to gesture-based commands has to be developed. A possible way to achieve the above goal is to exploit some type of reverse engineering-like technique, capable of making explicit the inner structure of applications' GUIs [28,29]. In particular, in the work described in [17], an approach originally designed to migrate a desktop-based application onto a mobile device is re-used to augment the interaction means available for controlling an existing application natively endowed only with traditional input capabilities [29]. The limit of [17] is that it is designed to handle only body gestures and, more importantly, that interaction is gathered by means of FAAST. Hence, the drawbacks regarding the method in [15] still apply.

Despite such efforts, a definitive integration layer capable of fully supporting the achievement of the above goals appears to be still unavailable. Thus, in this paper, a pluggable solution for improving the multi-modality of existing applications by adding personalized gesture- and voice-based control capabilities is presented.

In particular, the paper extends the work in [17] by removing the dependency on the mouse- and keyboard-mediated interaction of FAAST and by allowing the user to create a fully customizable library of poses, gestures and voice commands, thus effectively taking into account the needs that have been indicated among those possibly threatening or delaying a wider adoption of NUI solutions.

## 3. Designed Framework

**Figure 1.**
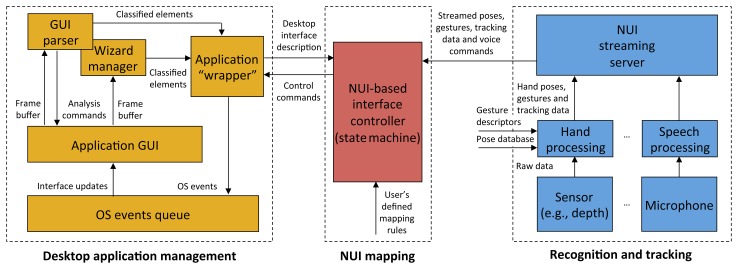
Overall architecture of the designed framework. The application “wrapper” communicates with the GUI parser/wizard manager to produce a description of the application's interface. The natural user interface (NUI)-based interface controller manages incoming poses, gestures, tracking data and voice commands transmitted by the NUI streaming server and, by acting as a state machine, translates them into control commands that are put into the events queue. The application interface is finally updated by the operating system to let the user appreciate the effect of his or her natural interaction.

The architecture of the designed framework is illustrated in Figure 1. Three conceptual blocks are proposed, dealing (from left to right) with the management of the application to be endowed with NUI-based interaction capabilities, the mapping of user's poses, gestures, tracking data and voice commands onto desktop-oriented commands tailored to such applications and, lastly, the extraction of hand and/or body tracking and speech data feeding the overall process.

In the following, the three blocks will be examined in detail. The discussion will consider first the desktop application management and the tracking blocks and will close by focusing on the mapping block, which actually plays a central role in the integration of the overall architecture.

### 3.1. Desktop Application Management

In the depicted schema, the management of the desktop application passes through the application “wrapper” component. The application wrapper is a type of “proxy” of the desktop application to be controlled. In a preparatory stage, it is responsible for generating the description of the application's GUI by means of the reverse engineering approach in [29]. In particular, this component can be used either to automatically extract a detailed description of the graphical elements constituting the interface or to support the users in the manual creation of such a description.

**Figure 2.**
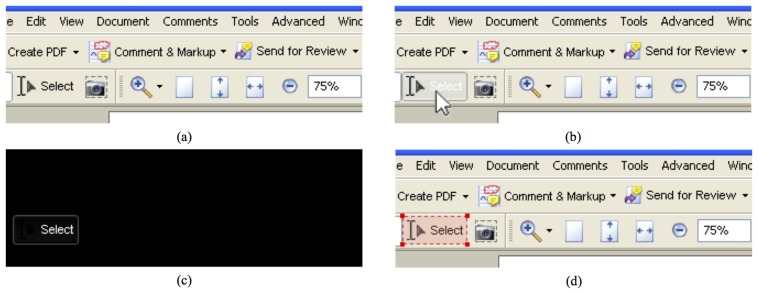
Steps of the process designed to identify and extract the elements constituting the application's GUI: (**a**) the portion of the interface before user interaction; (**b**) the appearance of the interface right after user interaction (mouse over the “select” button and the highlighting effect); (**c**) the difference image; and (**d**) the button identified.

In automatic mode, the application wrapper moves the mouse (through operating system calls) over imaginary horizontal lines spanning the entire interface, hence simulating a mouse-based user interaction. Interface elements react by changing their appearance or by forcing mouse pointer updates. The application wrapper continuously grabs the graphical content of the frame buffer just before and after the update. Differences identified by performing an exclusive OR between the captured images indicate the exact position of each particular interface element. As a matter of example, the application of the various steps of the above process on the part of the GUI for a common tool for viewing and editing .pdf files is illustrated in Figure 2.

**Figure 3.**
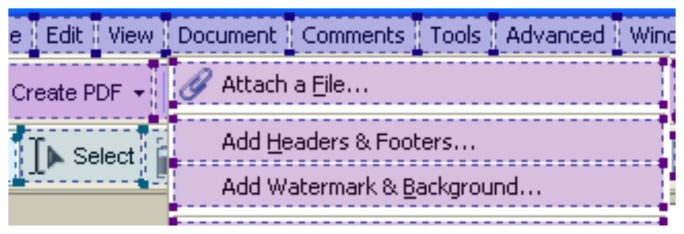
Graphical elements classified in the considered GUI (including menus, sub-menus, menu items, buttons, combo boxes, *etc.*).

Then, by means of suitable template matching rules, the application wrapper is able to classify each of the elements found as one of the interface components supported by the designed framework (including combo boxes, menus, sub-menus, menu items, buttons, text boxes and text areas, sliders, check boxes, scroll bars and other custom controls). As a matter of example, classification results obtained on the interface elements in Figure 2 are shown in Figure 3.

Once the graphical elements identified have been classified, a description of the interface is generated and stored in XUL (XML User Interface Language) format [30], a particular User Interface Description Language (UIDL) used to describe the visual appearance of a graphical interface as a set of structured elements. A video showing the overall process is available in [31].

**Figure 4.**
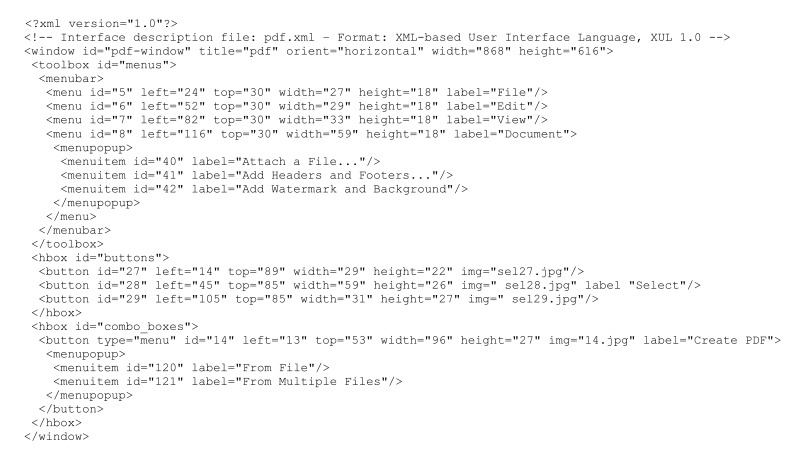
Portion of the XML User Interface Language (XUL)-based description for the considered GUI. For each element, the size and relative location in the desktop interface are reported.

In manual mode, the application wrapper provides the user with a set of wizards (which can also be used in automatic mode to improve element identification and classification accuracy) that let the user manually locate interface elements and assign them to a particular class. The XUL description file can also be extended/edited, e.g., to add hidden interaction commands that cannot be identified by automatic or wizard-based analysis. An excerpt of the XUL-based interface description for the considered application is provided in Figure 4.

### 3.2. Hand Pose/Gesture Tracking and Speech Recognition

The preparatory tasks described above have to be carried out just once for each application to be controlled. In fact, during normal operation, the application wrapper is designed to monitor and re-analyze the appearance of the application's interface to automatically identify dynamic changes that possibly occur during user interaction (opening of a dialog window, element switching from enabled to disabled, from visible to invisible, and *vice versa*, *etc.*).

The framework discussed in this paper needs to be fed with natural user interaction commands, which will be translated into desktop GUI events.

In [17], the interaction was based on FAAST and on a pre-defined set of body gestures, while movement in 3D space was used to control the mouse cursor position in the application's 2D space.

In this paper, a more general framework is designed, which is meant to let one or more NUI solutions to be used to enrich the interaction possibilities of desktop applications. In the following, attention will be specifically focused on hand poses/gestures and voice inputs. In particular, the Microsoft Kinect RGB-D sensor is used to develop a software component that is able to recognize different hand poses and gestures, as well as to provide continuous hand location and rotation information. At the same time, the Microsoft Kinect microphone is used to implement a speech recognizer. However, the framework has been designed by adopting a modular approach, which is expected to be capable of easing the integration of different hardware and/or software components tailored to other types of NUI-based interactions.

The designed hand tracking approach builds upon an extremely interesting pose estimation technique proposed by Oikonomidis *et al.* [32]. In the above work, the goal is to perform continuous finger tracking. Hence, the algorithm is designed to determine the angular position of every articulation in the hand skeleton for all of the possible hand movements at the cost of high computational complexity (the authors demonstrated their ability to work at nearly interactive frame rates only with a massively parallel implementation on a 1.581 GFlop GPU with 512 cores). Furthermore, the tracking method is not endowed with hand gesture recognition capabilities.

To relax hardware requirements and integrate missing functionalities, a number of optimizations are adopted in this paper by taking into account the specific role of the tracking system in the economy of the overall architecture proposed. A description of the inner details of the tracking system is out of the scope of this paper. Only the key modifications with respect to the work in [32] will be discussed.

**Figure 5.**
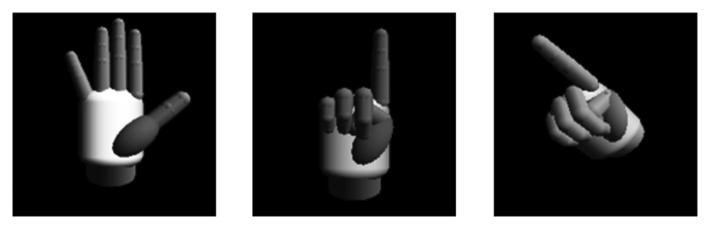
3D hand model used for generating the reference poses (three configurations are shown, obtained by working on a subset of the possible DOFs).

A key role in the optimization process is played by the newly introduced pose database, which contains reference hand poses to be recognized in visual data captured by the sensor during the interaction. These are “virtual” poses, because they are generated offline, starting from the 3D hand model displayed in Figure 5. Virtual poses are computed at system startup based on the dimensions of actual user's hands.

The model is characterized by 27 DOFs. After having defined the poses that the user wants to exploit for interacting with the applications and the possible movement ranges for each DOF, the pose database is populated with depth maps computed by rendering the model in all of the configurations required. While, in [32], all of the possible hand and finger configurations are configured online, in the optimized approach presented in this paper, the hand model is set up by adjusting all of the DOFs until just the set of possible poses and orientations of interest have been covered (some examples are reported in Figure 5). Moreover, in [32], poses are computed in real time starting from the previously-recognized hand configuration, whereas, in the proposed method, poses are generated at initialization and stored in the database. In the experimental tests that have been carried out, approximately 700 different configurations were generated for each basic pose (by applying rotations in steps of four to 15 degrees), resulting in thousands of depth maps stored in the database. A further optimization (simplification) consisted of down-sampling depth maps with the aim of reducing the number of points to be compared for pose recognition.

The optimized technique presented in this paper was proven to be able to carry out one thousand comparisons in nearly 20 milliseconds on a 3.2 GHz CPU.

**Figure 6.**
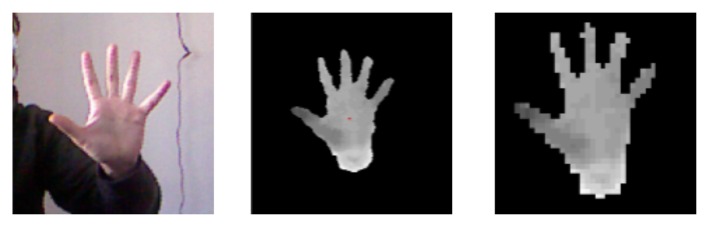
Hand segmentation, computation of the palm center and generation of the re-coded depth map to be used for querying the pose database: (**a**) image observed by the sensor; (**b**) segmented depth map; and (**c**) 40 × 40 pixel re-coded map.

The input of the hand-tracking module is a 320 × 240 pixel depth image. As illustrated in Figure 6, depth segmentation is used to isolate the hand in 3D. Only the pixels that lie in a defined depth range are kept, whereas the others are ignored. The largest region found is selected for further processing. In particular, to refine the outcomes of the segmentation step, depth pixels that are more than 10 cm from the previously determined palm center are discarded.

To compute a precise palm center, which is needed both to provide tracking data about the hand location and to carry out the above down-sampling, possible holes produced in the considered region need to be avoided. Hence, the region is first dilated by using a circular mask. Then, a rough evaluation of the area where the palm center is supposed to be is computed to speed up the process. Finally, the center is precisely identified through an accurate search within this reduced area. By resorting to the knowledge about the position of the user's hand, the initial image is re-coded into a 40 × 40 pixel depth map by cutting out all of the pixels without information and by scaling the hand region based on the real distance from the depth sensor. These steps guarantee that, independent of the position of the user's hand in the tracking space, consistent data for the pose estimation step is obtained.

**Figure 7.**
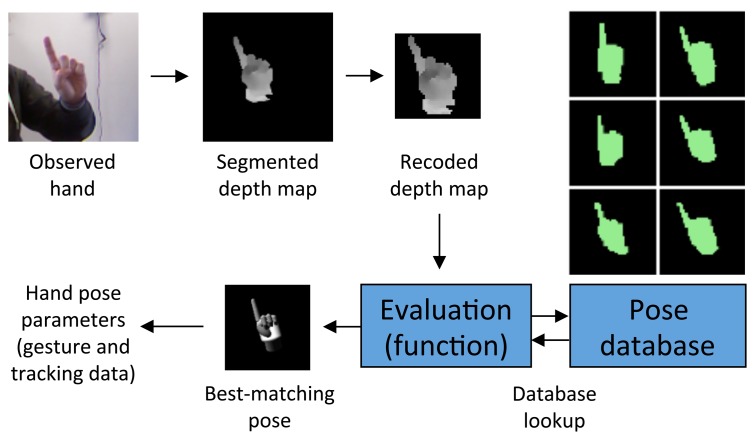
Hand pose estimation. The re-coded map containing the user's hand is compared against the pose database with an evaluation function working on depth distances. The configuration that is most similar is assumed as the estimate of the user's hand pose, and related parameters are extracted.

As shown in Figure 7, for each frame, the 40 × 40 pixel re-coded map is used to query the pose database with the goal of finding out the 3D rendered pose that is most similar to the current user's hand configuration. An evaluation function compares two depth maps by considering individual pixel values and returns a score that describes the distance between them. If the depths of two points are too different or one of the points is missing (depth value equal to zero), the function penalizes the score. Otherwise, it assigns a score that is proportional to the similarity of the depth values. Once the best-matching depth map has been found, associated parameters that were used for setting up the 3D model and generating the corresponding rendering immediately provide a complete description of the user's hand configuration. With the above process, it is also possible to track hand location and orientation in the current frame.

Starting from this information, previously processed frames are reconsidered to recognize the user's gestures. A simple filter is used to smooth rapid variations caused by possible errors in estimated poses. Recognition is then achieved by processing tracking information by means of a gesture descriptor-based classifier relying on the dynamic time warping (DTW) technique [33]. The DTW libraries for Microsoft Kinect were used [34]. The system is designed to recognize gestures, such as press, swipe, rotate, *etc.*, independently of the actual hand pose. Gestures are described in a 15-frame time window in terms of hand translations and rotations relative to the starting position and orientation. Recognition is performed on a 30-frame window, to manage gestures performed much more slowly than the reference ones. In the library, two thresholds are set on DTW-ed location data, one for activating gesture recognition and the second to actually recognize the gesture. The first threshold (for the maximum difference of the final hand position) was experimentally set to 8 cm. The second threshold (for the average difference of hand position throughout the gesture) was set to 5 cm. The use of rather strict thresholds reduced the number of false recognitions, making the classifier particularly robust. Recognition performances are studied in Section 4.

Pose and gesture recognition mechanisms are activated as soon as the hand calibration step has been performed (where the 3D model configuration to be later used for generating the pose database is determined by changing the dimensions of all of the hand parts and by computing the best match with the same evaluation function used in the recognition phase). Each time a frame is processed, the hand pose, location and rotation are determined. Moreover, for each frame, a gesture can additionally be identified.

Lastly, during hand tracking and pose/gesture recognition, a voice command can eventually be identified by the speech recognition module, which has been implemented using the Microsoft Kinect SDK and integrated into the overall architecture.

Pose, gesture, tracking data and voice commands are made available by means of a streaming server. The server can be accessed over a network socket. Communication is based on a simple application-layer protocol built upon plain text messages. Each message can contain information about hand location and orientation, current pose, recognized gesture or voice command. In the devised architecture, streamed data are transmitted to the NUI-based interface controller, where it is mapped to the user's customized commands for controlling the application's GUI. It is worth observing that, thanks to the use of a streaming server, the machine hosting the sensors and running the tracking and recognition software does not necessarily have to be the same machine that is running the application to be controlled.

### 3.3. NUI Mapping

The responsibility for the integration of the desktop application management module and the recognition and tracking block is on the NUI-based interface controller, which is in charge of translating pose, gesture and voice commands into GUI events.

Thus, when the user selects the particular application to interact with, the application wrapper delivers the corresponding interface description to the NUI-based interface controller, which allows the user to specify a mapping between poses/gestures/voice inputs and control commands targeted to specific interface items. The mapping for the given user/application is stored for possible re-use at a later time. Once a mapping has been defined, the NUI-based interface controller connects to the NUI streaming server and maps incoming messages onto the corresponding control commands. Such information is delivered to the application wrapper module, which is responsible for translating it into suitable instructions to be inserted into the events queue. Events will be then processed by the operating system and will produce the expected effect on the application, thus closing the overall control loop.

**Figure 8.**
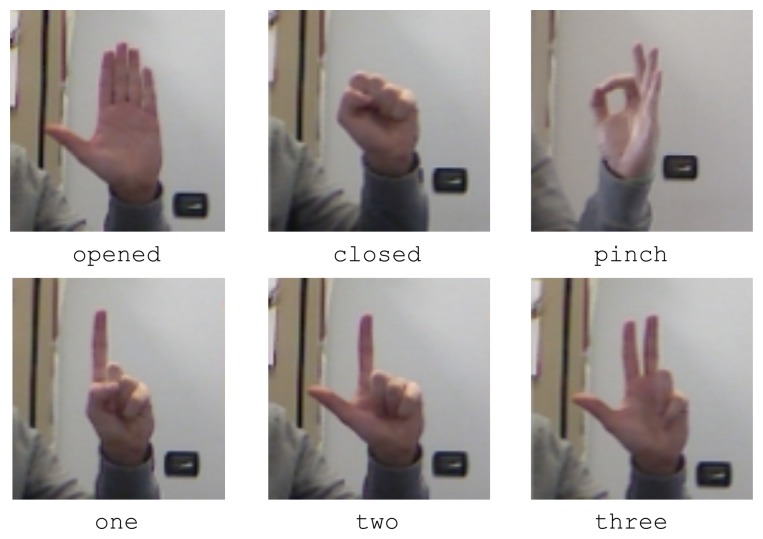
The six users' poses (RGB data) considered in the experimental tests.

At present, a protocol based on raw text is used (although work is in progress to switch to a JSON-based one to improve extendability). Messages delivered by the steaming server are separated by a newline character. The space character is used to tokenize elements on any given line. Each message begins with a code describing the message type. When a frame is processed, the server sends a POSE message describing the position and orientation of the user's hand in the format:

POSE name x_pos y_pos z_pos x_rot y_rot z_rot

where the x_pos, y_pos and z_pos values represent the spatial (world) coordinates in meters, x_rot, y_rot and z_rot are the rotation angles along the three axes and name is the name of the basic pose recognized. In the experiments performed, the six basic poses reported in Figure 8 were considered: opened, closed, pinch, one, two and three (fingers).

If, at a certain frame, a gesture is also recognized, the server sends out a GESTURE message, whose format is:

GESTURE name

where name indicates the gesture found. At the present time, the designed tracking system has been endowed with descriptors for recognizing the following gestures: press, scroll, rotate_cw, rotate_ccw. left_swipe. right_swipe, up_swipe and down_swipe. Given the modular approach pursued, it would be quite easy to extend the current set of basic pcees and gesture descriptors to embed technologies and algorithms capable of recognizing a larger set of interaction commands (e.g, gathered by a different device, such as the Leap Motion 3D Controller).

The above gestures are described independently of the particular pose. That is, the user could make the press gesture both with his or her hand opened or closed Nonetheless, to simplify the implementation of the NUI-based interface controller, several higher-level gestures corresponding to a switch from one pose to another have been implemented. For instance, the system recognizes an opening gesture when the user opens his or her hand (similarly for the closing gesture), a pointing gesture when the pose is changed to one (corresponding to the index finger) to indicate something in the scene, *etc.*

Lastly, when a voice command is recognized, the server generates a VOICE message, with the same format of the GESTURE message. In this case, name indicates the input recognized.

Otter interaction commands, e.g. collected by otter sensors, could be added, by re-using the above messages or defining new ones.

The user is allowed to setup basic one-to-one mappings by assigning tracking information contained in the above messages to a particular graphical element. Thus, for instance, hand location information could be used to move the mouse pointer over a particular button of the interface. Then, a press gesture or a “press” voice command could be used to activate it. This would enable a trivial translation from a mouse-based interaction paradigm to a NUI-based one.

However, there are situations where one-to-many combined mappings might be needed [35]. In fact, the real advantages associated with the adoption of the NUI-based interaction may be fully appreciated when the basic paradigm is replaced, or at least accompanied, by a new approach, where a given task that is hard to accomplish in the traditional way (e.g., because it requires many mouse-keyboard interactions) can be carried out by means of a simple and customizable command. This is the case, for instance, of an application functionality activated by the selection of a sub-menu item. With the one-to-many mapping functionality introduced in the designed framework, the action of moving the mouse over the menu, performing a click on it, waiting for its expansion, scrolling through sub-menu items and finally performing a click on the entry of interest could be replaced by (combined into) a single pose, gesture or voice command. It will then be the task of the application wrapper to translate it into separate events to be pushed in the operating system queue.

## 4. Evaluation of Performances

Experimental evaluation has been carried out by focusing on three perspectives. Firstly, tests were performed to measure the performances of the pose and gesture recognition systems. Secondly, experiments were carried out to analyze how the framework illustrated in this paper could be configured to add new interaction possibilities to existing applications. Thirdly, the study served to carry out a preliminary evaluation of the advantages that could be obtained with (and the drawbacks that could result from) the introduction of natural interaction into desktop applications. The results for the first perspective will be presented below. Findings for the other two perspectives will be discussed in Section 5.

### 4.1. Performances of Hand Pose Recognition

The pose recognition system used in this paper has been developed starting from the technique in [32], which was not meant to perform such tasks and required a GPU to work at nearly interactive frame rates. Hence, in the following, a characterization of the robustness and computational complexity of the adapted algorithm (as discussed in Section 3.2) is presented.

Robustness has been evaluated by measuring the probability of a pose to be confused with other poses. Tests were performed on a 3.2-GHz CPU, with a pose database generated starting from the basic poses illustrated in Figure 8, *i.e.*, opened, closed, pinch, one, two and three. In each test session, the user was asked to perform the calibration step and then move and rotate his or her hand in all of the possible directions while keeping a single pose. Translation and rotation data along the three axes were recorded for over 1000 frames. The test was performed for all of the poses in the set and repeated by five subjects (four male and one female researchers in the 25–30 age range working in the Graphics and Intelligent Systems/Ubiquitous Computing Lab at Politecnico di Torino). The results obtained are displayed in the confusion matrix reported in Table 1.

It is worth observing that, in the scenarios of interest for this paper, the number of poses required may change from one application to another. Hence, the tests above were repeated by changing the configuration of the pose database, *i.e.*, by varying the number and types of poses to be recognized (e.g., from {opened, closed}, to {opened, pinch}, {opened, pinch, closed}, *etc.*). For the sake of compactness, the results are illustrated in Table 2 in terms of correct recognition rates. The first column reports the number of basic poses used to generate the virtual depth maps stored in the database. The next columns report correct recognition percentages obtained when the particular pose indicated in the column header was included in the set (for the various set sizes considered). Starting with a set of two poses, the various combinations of the six basic poses have been studied. The last column reports the average results.

It can be observed that the overall recognition rate decreases with the size of the set. By augmenting the number of poses in the set, the error rate grows, primarily because of the presence of poses that differ slightly from one another (e.g., poses one and two). It is easy to see that, in a set with two poses, the choice guaranteeing the highest robustness would be {opened, closed}. In a set of three poses, it would be preferable to choose {opened, closed, pinch}.

Regarding computational complexity, each depth map produced by the sensor took nearly 50 milliseconds to be processed. Hence, the optimized pose recognition algorithm is able to guarantee an average tracking speed of roughly 20 frames per second on the CPU selected in this paper.

### 4.2. Performances of Hand Gesture Recognition

An approach similar to the one adopted to characterize the pose recognition algorithm was pursued to measure the robustness of the gesture recognition system. In this case, the five subjects involved in the first test sessions were requested to repeat each gesture 100 times while keeping the same pose (specifically, the opened one). Robustness was evaluated by measuring the probability of a gesture to be confused with other gestures and by determining, for each gesture, the overall and correct recognition rates. The sample size was determined by setting a confidence level of 95% and a margin of error of 5% for recognition results. The results are reported in Tables 3 and 4, respectively.

From Table 3, it can be easily observed that the thresholds used in the DTW made the algorithm quite robust, at the cost of a rather high number of times for which the system does not recognize any gesture (second column of Table 4). It is worth observing that, although the system is designed to work in a pose-independent way, recognition rates may vary based on the actual pose used. Hence, a more accurate characterization may be required, e.g., to automatically choose gestures to use for controlling a given application based on robustness criteria.

## 5. User Study

By leveraging the results obtained in the above experiments, further experimental tests were carried out with the aim of studying the possible benefits coming from the integration of natural interaction modalities with desktop applications natively developed to be operated via the traditional mouse-keyboard paradigm. The tests were organized into two experiments. The first experiment focused on the hand pose/gesture-based interaction. Based on the results gathered, a second experiment was designed to evaluate the effect of multi-modality by adding voice-based interaction possibilities.

Qualitative and quantitative observations were collected by means of a user study that involved 30 volunteers (23 males and seven females) selected among the students and teachers of various courses for engineering and architecture degrees at Politecnico di Torino. The data collection met the requirements of the Ethical Framework of the University, and informed consent was obtained by all individuals. Volunteers were asked to carry out several tasks by working on two particular applications that were selected based on the richness of the graphical interface, the variety of interaction possibilities and the complexity of tasks that could be accomplished. Tasks were performed both with the native and the natural interface to analyze the impact of new interaction possibilities (added by either using the framework illustrated in this paper or coding/re-coding the application's interface).

A default mapping between natural inputs and interface control commands (namely, the one ensuring the highest robustness) was used for all of the volunteers, with the aim of making the results obtained with the two interaction modalities comparable.

### 5.1. Applications Selected for the Experiments

**Figure 9.**
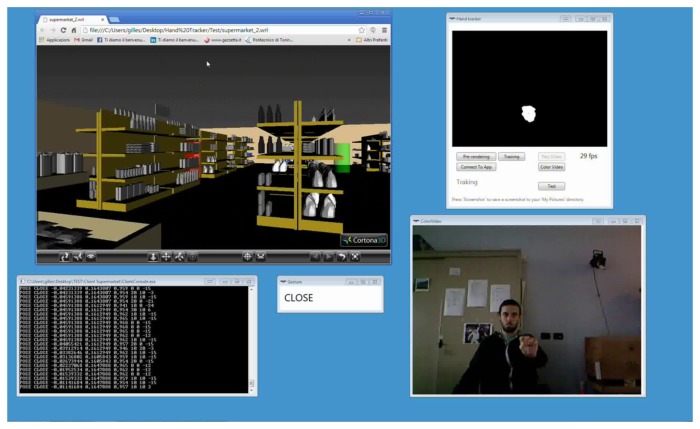
Screenshot of Cortona3D Viewer during experimental tests.

**Figure 10.**
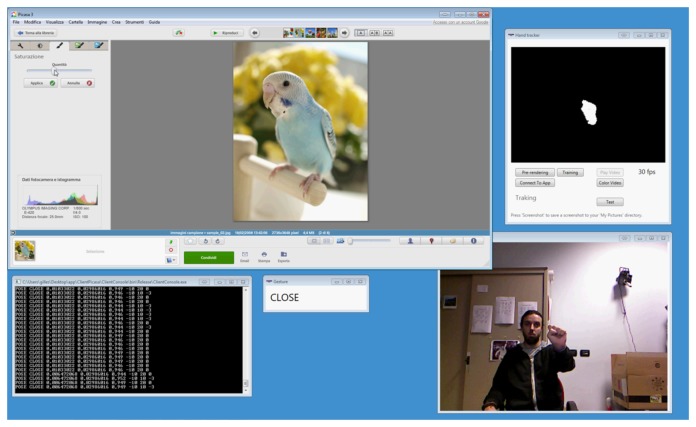
Screenshot of Google Picasa during experimental tests.

The two applications selected were Cortona3D Viewer [36] and Google Picasa [37]. Cortona3D Viewer can natively be used to load and navigate Virtual Reality Modeling Language (VRML)-based virtual scenes by means of simple mouse and/or keyboard commands. Google Picasa allows users to organize, view, edit and share photos on the web. Two screenshots showing user interaction with the above applications (depicted in the top left) are presented in Figures 9 and 10.

The application wrapper component was first exploited to generate a description of graphical elements embedded in the interfaces of the two applications. For instance, for the Cortona3D Viewer, the controls available are mainly aimed at letting the user move, rotate and scale the models displayed, as well as move the camera around the scene. By means of the framework illustrated in this paper, the application interface could be provided with natural control possibilities. For instance, the user might choose to setup the mapping by exploiting three hand poses, namely opened, closed and one, as well as the down_swipe gesture. With the opened pose, the mouse could be moved over the screen without affecting the application's behavior. With the closed pose, the user could decide to move the camera or the model. With one, the model could be brought back to its default configuration. By moving the hand towards the sensor (or retrieving it), a zoom in (or zoom out) could be performed. By making a down_swipe gesture with the opened pose, the user could switch between translate and rotate modes. A video showing the interaction with a sample mapping, such as the one described above, is available in [38].

A comparable procedure was applied to the interface of Google Picasa. In this case, multi-modal inputs might be used, for instance, to navigate a photo gallery, pick a photo out of it and apply a number of transformations and filters.

### 5.2. First Experiment: Hand Pose and Gesture-Based Interaction

The first batch of tests was aimed at measuring the effectiveness of application control by means of hand poses and gestures. Two scenarios were set up to study the possibilities offered by one-to-one and one-to-many mapping rules.

#### 5.2.1. Setup of the Interaction Scenarios

The first scenario for this experiment consisted of exploring a 3D supermarket environment [39] with Cortona3D Viewer while searching for five objects placed in as many fixed (but unknown) positions (Figure 9). The test was considered completed when all of the objects had been collected. The objects were distributed all around the supermarket, to force the users to interact with the application for roughly one to two minutes. Two videos showing mouse-keyboard [40] and the hand pose/gesture-based interaction are available [41]. In this case, a one-to-one mapping of a very limited set of mouse-keyboard events onto equivalent hand poses and gestures was tested. In particular, the proposed framework was configured to map the movement of the closed hand (closed pose) in the user's 3D space on native mouse-keyboard commands for exploring the virtual environment.

For the second scenario, the editing of eight photos with Google Picasa was required by picking them out from a gallery and making modifications (Figure 10). In particular, for six of the photos, users were requested to set the saturation to zero. For the third and fifth photo, users were asked to cut out a portion of the scene and apply a color inversion filter to it. A video showing user operation with the native interface is available in [42]. As can be observed, the user interaction is quite intense, requiring the user to perform a number of operations to select the right editing tool and use it on the photo. In this case, a one-to-one mapping was combined with more complex mapping rules by associating given poses or gestures with a sequence of user interface events. Thus, for instance, the act of pushing the open hand toward the screen (press gesture and opened pose) was mapped to the photo selection operation, whereas the horizontal swipe (left_swipe or right_swipe gesture) was used to move to the next photo. However, the mapping was also configured in such a way that, for instance, by closing the hand (closed posed) and moving it to the left/right, the color saturation tool was automatically selected, the actual saturation value was decreased/increased by operating the corresponding slider, and the modification was applied to the photo by activating the confirm button. Similarly, the cutting tool was activated by the pinch pose, while the up_swipe gesture allowed the users to directly apply the color inversion filter. A video showing the hand pose/gesture-based interaction with the above mapping is available in [43].

#### 5.2.2. Hypotheses

Based on design objectives and rough feedback collected in the performance evaluation steps, the following hypotheses were formulated for the experimental tests with the two scenarios above.

- H1. The average time needed to explore the supermarket with Cortona3D Viewer by using the hand pose/gesture-based method is lower than or comparable to that required by using the native mouse-keyboard interface.
- H2. The average time needed to complete the photo editing task with Google Picasa by using the hand pose/gesture-based method is lower than that required by using the native mouse-keyboard interface.
- H3. Intuitiveness in the exploration of the supermarket with Cortona3D Viewer by using the hand pose/gesture-based method is comparable to that experienced by using the native mouse-keyboard interface.
- H4. Intuitiveness in performing the photo editing tasks in Google Picasa by using the hand pose/gesture-based method is comparable to or higher than that experienced by using the native mouse-keyboard interface.
- H5. Fatigue is higher with the hand pose/gesture-based method than with the native mouse-keyboard interface for both the Cortona3D Viewer and Google Picasa scenarios.

On the one hand, when a direct mapping strategy is adopted, hand poses and gestures are basically used as a straightforward replacement of the mouse and keyboard. Hence, no dramatic savings (in terms of time) or improvement (in terms of intuitiveness) should be expected. On the other hand, when combined mapping is used, a higher number of time-consuming operations on the graphical user interface can be linked to a single pose and/or gesture. Thus, lower completion times and comparable or even higher intuitiveness could be observed. However, complaints about the higher physical effort required by hand/arm-activated interfaces are expected, as well.

#### 5.2.3. Participants, Procedure and Variables Measured

The user population was composed of 20 volunteers in the age range from 20 to 40 years old. Given their age and background, almost all of the end-users already knew how to use 3D and graphical applications, such as the ones selected for the study. In this way, the possibility of having results biased by the difficulty of learning a new graphical user interface was reduced. All of them were used to carrying out common digital tasks, such as reading email, surfing the web, *etc.*, by using a touch-based device, and they were very satisfied with it. Eighteen already used their voice to carry out some tasks, though their opinions about such interaction means were not particularly positive. Nine had already experienced body gesture-based interaction, mainly from playing video games on a console, with an intermediate degree of satisfaction. Only three out of twenty users had experienced hand gesture-based interaction before the test. As their performances did not stand out, they will be included in the main population in the following, and their results will be processed together with those of the other end-users.

Each user was individually trained on the execution of the tests. After a brief familiarization with the applications and the interaction means, each user was requested to complete the two tests, both with the native interface and with the hand pose/gesture-based one. The tests were submitted in a random order. Half of the population started the test with the native interface and then switched to the hand/gesture-based one. The other half started with the hand/gesture-based interface to counterbalance the experiment. For each test, every user was asked to perform three trials. The time for the completion of each trial was recorded. After completing the tests, users had to fill out a questionnaire in two parts.

In the first part, for both of the scenarios, the user was asked to confirm the suitability of the mapping adopted and to rank the intuitiveness of the mouse-keyboard and hand pose/gesture-based interaction modalities. Intuitiveness was described in terms of familiarity, *i.e.*, as an indication of how comfortable the user felt with the particular interface after having been briefly instructed on how to use it and having performed the particular task [44]. The user was also requested to indicate the interaction modality that was preferred overall (choosing among mouse-keyboard, hand poses/gestures and no preference).

In the second part (based on ISO9241-9 and devoted to device assessment [45]), the user was asked to answer specific questions about the actuation force required, operation smoothness and speed, mental and physical effort, accuracy, fatigue and general comfort. No distinction was made between the two scenarios, and responses for the mouse-keyboard and hand pose/gesture-based interaction were recorded on a seven-point Likert scale.

#### 5.2.4. Results

**Figure 11.**
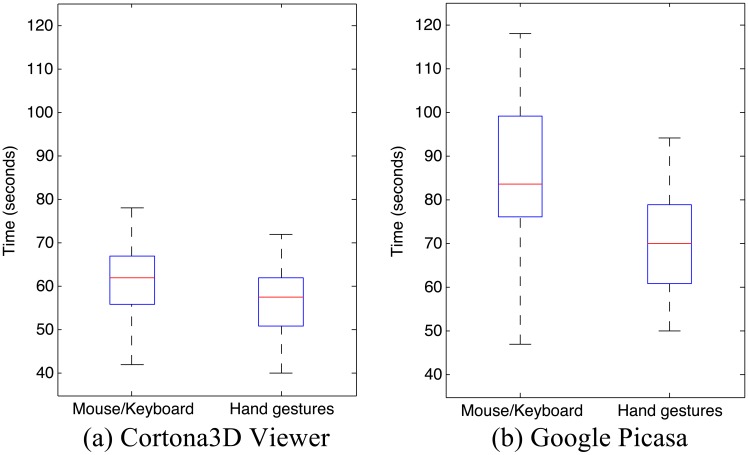
Box plots of completion times by interaction technique in the (**a**) Cortona3D Viewer and (**b**) Google Picasa scenarios.

Results pertaining to completion time are reported in the box plots in Figure 11. It can be easily observed that, for both the tests, average completion time with the hand pose/gesture-based interface is lower than with the native one.

In particular, in the first scenario (Figure 11a), the average completion time was 61:23 s (*σ*^2^ = 8.50) with the mouse-keyboard and 56.73 s (*σ*^2^ = 7.95) with the hand pose/gesture-based interface. As expected, the difference was even more marked in the second scenario (Figure 11b), with an average completion time of 85.86 s (*σ*^2^ = 16.69) and 70.68 s (*σ*^2^ = 12.23) with the mouse-keyboard and the hand pose/gesture-based interfaces, respectively. The statistical significance of the results was assessed by using paired *t*-tests and a level of significance of *α* = 0.05. Because of the values of *p* = 0.008 and *p* = 0.0002 for the first and second test, respectively, the null hypothesis could be rejected, confirming the validity of both H1 and H2.

**Figure 12.**
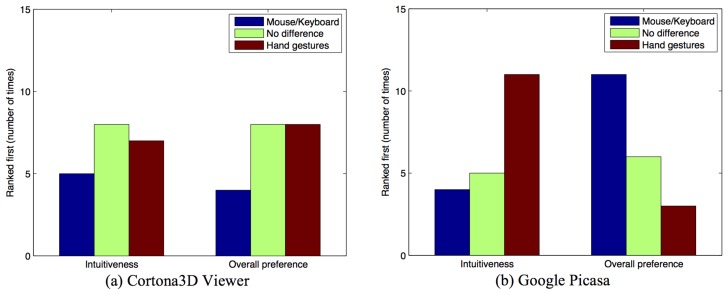
Number of times a given interaction techniques has been indicated as the most intuitive or preferred, overall, in the (**a**) Cortona3D Viewer and (**b**) Google Picasa scenarios.

Results obtained from the questionnaire concerning the perceived intuitiveness and preferred interaction modality are reported in Figure 12. For the first scenario (Figure 12a), employing the Kruskal–Wallis h-test, we could not find a statistical significance for intuitiveness (*χ*^2^ = 1.032, *p* = 0.597) or for personal preference (*χ*^2^ = 2.36, *p* = 0.307). On the contrary, in the second scenario (Figure 12b), participants considered the hand pose/gesture-based interface as the more intuitive (*χ*^2^ = 6.342, *p* = 0.042), thus validating H4. However, they preferred the mouse-keyboard interaction overall (*χ*^2^ = 7.228, *p* = 0.027), though it is easy to observe that the number of times the users preferred hand poses and gestures or had no preference is comparable to the number of times the mouse-keyboard interaction modality was chosen.

**Figure 13.**
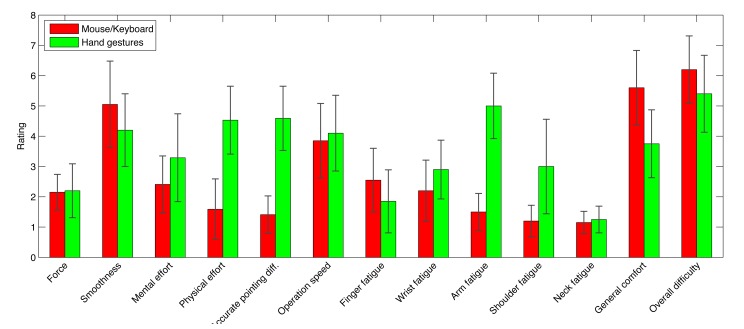
Average ISO90241-9 ratings for the two interaction device on a seven-point Likert scale (error bars indicate the standard deviation).

The findings above can be better understood by considering also Figure 13, where the average ratings assigned to questions based on ISO90241-9 are reported.

Here, no distinction is made between the two scenarios. By focusing on relevant (and statistically significant) results, it can be observed that the hand pose/gesture-based interface required a higher mental effort and provided less smoothness. Accurate pointing was much more difficult to achieve with hand poses and gestures than with the mouse and the keyboard. Moreover, hand poses and gestures resulted in significantly higher wrist, arm and shoulder fatigue, making the overall comfort much lower than that of mouse-keyboard-based interaction (thus confirming H5).

Taking into account the scope of ISO90241-9 and that all of the users, but one, confirmed the adequateness of the mapping strategy adopted, the results above regarding personal preference could be reasonably linked to various particular features of the scenarios considered and to the possible limitations of the interaction device adopted. For instance, mental effort could be due to the complexity of remembering the (possibly too large) set of poses/gestures chosen to control the application's behavior, whereas physical effort is clearly associated with the use of a body sensor for performing hand tracking. Pointing accuracy is limited by the characteristics of the sensor used, and its impact is particularly critical for tasks requiring precise point-and-click interaction (such as cutting a photo). Therefore, it is possible that the time savings obtained in the Google Picasa scenario were not able to counterbalance the poor usability performances (which, in that scenario, might be particularly critical).

### 5.3. Second Experiment: Multi-Modal Interaction

From the results obtained with the two scenarios considered in the first experiment, it was evident that defining a hand pose/gesture-based mapping for all of the commands of a common desktop application could result in an extremely complex interaction mechanism. As an example, it would be very difficult to map not only saturation, but all of Google Picasa's tools for controlling image contrast, brightness, hue, *etc.* Indeed, this observation confirms the fact that natural interaction means would probably have to be considered as a method of enriching existing interaction mechanisms, rather than replacing them. Nonetheless, part of this complexity may be because hand- and gesture-based interaction alone does not provide the degree of flexibility that could be achieved in a richer multi-modal scenario.

With the aim of checking the validity of this hypothesis, a way to manage a situation, such as the one depicted above for Google Picasa, was studied. A possibility could be, for instance, to use mouse-keyboard and hand poses/gestures together. However, the switch between the two interaction modalities could be quite hard to manage. It was therefore decided to experiment with pose- and gesture-based interactions combined with voice commands.

#### 5.3.1. Setup of the Interaction Scenario

In the second experiment, a third interaction scenario was set up. The test consisted of again editing eight photos with Google Picasa by repeating some of the operations in the second experiment, but also working with several editing tools not yet explored. A video showing user operation with the mouse-keyboard interface is available in [46]. As can be observed, eight different editing tools are used (e.g., for defocusing the image, cutting it, applying the film grain or posterization effects, *etc.*), which are accessed through various tabs of the interface. Once activated, a given tool is controlled by a number of (repeated) graphical widgets, such as buttons and sliders. To address the issues found during the previous experiment, pose/gesture-based tool selection was replaced by voice commands. Thus, for instance, the defocusing effect was activated by issuing the “defocus” or “defocusing” command. In this way, a larger number of application functionalities could be activated. Then, a limited, intuitive and (hopefully) easy-to-remember set of hand poses and gestures was mapped onto the above widgets. For instance, when moved horizontally with the closed pose, the user's hand controlled the first slider in the tool panel; when moved vertically, it controlled the second slider, and so on. The pinch pose could be used to cut a photo, whereas a horizontal swipe was used to change the photo to be edited. A video showing multi-modal interaction with the above mapping is available in [47].

#### 5.3.2. Hypotheses

In the third scenario, the goal was to study the effect of complementing hand pose- and gesture-based interaction with voice commands. Because limitations for the pose- and gesture-based interface, e.g., in terms of accuracy and physical effort, had already been determined in the previous experiments, the analysis for the current scenario was focused mainly on operation speed, mental effort and intuitiveness. In particular, the following hypotheses were formulated.

- H6. The average time needed to complete the photo editing task with Google Picasa by using hand poses/gestures and voice commands is still lower than that required by using the native mouse-keyboard interface.
- H7. Mental effort to perform the photo editing tasks with Google Picasa by using hand poses/gestures and voice commands is now lower than that experienced by using the native mouse-keyboard interface.
- H8. Intuitiveness to perform the photo editing tasks with Google Picasa by using hand poses/gestures and voice commands is higher than that experienced by using the native mouse-keyboard interface.

#### 5.3.3. Participants, Procedure and Variables Measured

For the third scenario, 10 new volunteers were selected, to not have results biased by previous experience with the framework. Volunteers showed almost the same characteristics of those selected for the experiments with the first two scenarios. In particular, almost all of them already used their voice to carry out some tasks. Only two of them had experienced hand pose/gesture-based interaction before the test. Each user was individually trained on the execution of the task, using both the mouse-keyboard and the multi-modal interfaces. Then, half of the users were requested to carry out the task with the mouse-keyboard interface and then to switch to the other interaction method. For the other half, the order was inverted. For each test, every user was asked to perform two trials. The time for the completion of each trial was recorded, together with the number of times the user issued a wrong pose/gesture or voice command. After completing the tests, users had to fill out a questionnaire that was much shorter than the one used for the first two scenarios. In fact, they were asked to evaluate only the mental effort and intuitiveness of the two interaction modalities, also by indicating the preferred one.

#### 5.3.4. Results

The results pertaining to completion time confirmed that multi-modal interaction, the same as the pose- and gesture-based one (alone), can produce a significant speedup over the natural interface (in this case, due partly to the one-to-many mapping and partly to the use of voice-based “shortcuts”). In particular, the average completion time was 178.70 s (*σ*^2^ = 22.63) with the mouse-keyboard interface and 145.90 s (*σ*^2^ = 26.18) with the multi-modal interface. The statistical significance of the results was again assessed by using paired *t*-tests. A value of *p* = 0.0019 was obtained, rejecting the null hypothesis and confirming the validity of H6.

Mental effort was rated as 3.30 (*σ*^2^ = 1.16) and 1.61 (*σ*^2^ = 0.84) with the mouse-keyboard and the multi-modal interfaces, respectively. The statistical significance was tested using the Kruskal–Wallis h-test (*χ*^2^ = 8.758, *p* = 0.0031), validating H7. An additional confirmation of the reduced mental effort obtained with the multi-modal interaction modality came from the number of times the user issued the wrong command using any of the interaction modalities available, which was only 1.21 on average.

Regarding intuitiveness, seven (one) users indicated the multi-modal (mouse-keyboard) interface as the more intuitive, whereas two of them had no preference (*χ*^2^ = 8.99, *p* = 0.011), validating H8. Moreover, six users indicated the multi-modal interface as the preferred one overall, whereas no one chose the mouse-keyboard interface and four had no preference (*χ*^2^ = 8.12, *p* = 0.017).

### 5.4. Considerations and Remarks

The user study confirmed that, in the scenarios considered, the introduction of multi-modal control possibilities can improve efficiency by reducing interaction time, especially when multi-modal commands are used to replace a sequence of mouse-keyboard operations. The more frequent and time-consuming operations are combined via a one-to-many mapping, the higher the time savings expected could be. The results obtained in the first experiment also showed that, for simple interaction tasks, a clear preference for a particular interface could not be identified. On the contrary, when dealing with complex interactions, hand poses and gestures are considered not only more effective, but also more intuitive than the mouse-keyboard. However, the impact of such advantages could be reduced by the poorer user experience due to higher physical and mental effort, which are experienced, e.g., when the hand- and gesture-based interaction is used alone.

In fact, the second experiment showed that, by introducing suitable mechanisms to reduce the mental effort, e.g., based on voice commands, the intuitiveness of the hand pose- and gesture-based interaction can be further improved, making it the preferred interface for carrying out the tasks selected for the experiments.

Hence, several guidelines could be drafted to mitigate the above constraints and foster the exploitation of natural user interaction modalities in desktop applications. For instance, hand pose- and gesture-based interaction devices achieving better scores in ISO90241-9 terms should be selected. As a matter of example, the Leap Motion 3D Controller or the Myo armband by Thalmic Labs [48] could allow for reduced physical effort. Moreover, mental effort could be addressed by introducing a multi-layer mapping, such as the one exploited in the last scenario considered. In this case, the first interaction mechanism was used to activate a given set of application functionalities, whereas the second mechanism was exploited to define a limited set of commands to be re-used several times for different purposes. Furthermore, in the definition of the pose/gesture set and mapping rules, robustness considerations should be accompanied by usability criteria (to make the above set as easy to remember as possible), by possibly allowing the user to configure the preferred mapping (as could actually be done in the presented framework, if needed). Lastly, the negative impact of accuracy could be limited by properly separating application functionalities to be handled by means of natural interaction from those that should continue to be managed through the native interface.

## 6. Conclusions and Future Work

In this paper, a framework aimed at extending the applicability of NUI-based interaction techniques to existing desktop applications without any code re-writing effort is presented.

The proposed approach exploits *ad hoc* image processing techniques to unveil (and access) the inner structure of the application's GUI. Once the structure of the interface has been identified, user's poses, gestures and voice commands can be easily linked to graphical elements found by means of customizable mapping rules. With respect to other comparable solutions reported in the literature, the devised approach does not rely on the existence of specific keyboard- or mouse-based control functionalities (or shortcuts) in the original interface and allows the user to create a library of personalized interaction controls.

The framework has been tested with a dedicated hand-tracking component based on a gaming RGB-D sensor and with a speech recognition module based on standard libraries, though it has been designed in a modular way to be easily extended for managing other sensors and recognition solutions. For instance, at present, work to integrate a module for getting hand/finger tracking data from the Leap Motion 3D Controller is in progress.

Experimental measures carried out to evaluate both tracking performance, as well as application control functionalities enabled the identification of several criteria for an effective integration of the hand pose- and gesture-based interaction into desktop applications. A user study in which 30 volunteers were asked to test the proposed framework with two selected desktop applications showed that more relevant time savings and improved intuitiveness can be observed when a single hand pose/gesture-based interaction is used to control several time-consuming operations on the target interface. The above benefits are balanced by a higher physical and mental effort required by the hand/arm-activated interaction, which also results in less smoothness and poorer accuracy. Nonetheless, pose/gesture- and voice-based multi-modality might help to reduce mental effort, by further boosting time performances and intuitiveness.

Future works will be aimed at increasing the interaction means supported by the framework, e.g., by integrating eye gaze tracking techniques, brain stimuli, *etc.*, generally available only as separate features. Moreover, additional tests will be carried out on an extended set of application by also experimenting with the system's personalization features with the aim of defining application- and user-specific poses, gestures, voice commands and mappings capable of guaranteeing an improved user experience. Lastly, there are plans for experimenting with the applicability of the proposed framework in other scenarios, e.g., in the industrial and service robotics fields.

## Figures and Tables

**Table 1.** Confusion matrix for the pose dataset in Figure 8.

	**Open**	**Closed**	**Pinch**	**One**	**Two**	**Three**
open.	95.7	2.8	0.0	0.0	0.0	1.5
closed	0.0	94.6	0.0	5.4	0.0	0.0
pinch	7.8	6.4	85.8	0.0	0.0	0.0
one	0.0	21.3	0.0	71.9	6.8	0.0
two	0.0	2.5	0.0	19.8	77.7	0.0
three	0.0	0.0	0.0	9.5	6.3	84.2

**Table 2.** Recognition rates (percentages) obtained for pose set sizes from two to six by changing poses in the set.

**Pose Set Size**	**Open**	**Closed**	**Pinch**	**One**	**Two**	**Three**	**all**
2	98.9	96.8	96.4	93.7	95.5	96.6	96.3
3	97.4	95.9	93.0	86.5	89.9	92.8	92.6
4	96.7	95.2	90.2	80.1	84.0	90.5	89.5
5	96.1	94.9	87.3	75.8	80.2	87.7	87.0
6	95.7	94.6	85.8	71.9	77.7	84.2	85.0

**Table 3.** Confusion matrix for the gestures recognized by the system as given in Section 2.

	**Left_Swipe**	**Right_Swipe**	**Up_Swipe**	**Down_Swipe**	**Rotate_cw**	**Rotate_ccw**	**Press**
left_swipe	98.6	0.0	0.0	0.0	0.0	0.0	1.4
right_swipe	0.0	99.3	0.0	0.0	0.0	0.0	0.7
up_swipe	0.0	0.0	88.3	0.0	0.0	0.0	11.7
down_swipe	0.0	0.0	0.0	87.0	0.0	0.0	13.0
rotate_cw	0.0	0.0	0.0	0.0	100.0	0.0	0.0
rotate_ccw	0.0	0.0	0.0	0.0	0.0	100.0	0.0
press	0.0	0.0	0.0	0.0	0.0	0.0	100.0

**Table 4.** Overall recognition rates and correct recognition rates (percentages) for the gestures reported in Section 2.

**Gesture**	**Overall Recognition**	**Correct Recognition**
left_swipe	87.2	86.0
right_swipe	85.0	84.4
up_swipe	66.8	59.0
down_swipe	75.4	65.6
rotate_cw	85.2	85.2
rotate_ccw	83.4	83.4
press	66.6	66.6
